# Towards the Growth of Hexagonal Boron Nitride on Ge(001)/Si Substrates by Chemical Vapor Deposition

**DOI:** 10.3390/nano12193260

**Published:** 2022-09-20

**Authors:** Max Franck, Jaroslaw Dabrowski, Markus Andreas Schubert, Christian Wenger, Mindaugas Lukosius

**Affiliations:** 1 IHP—Leibniz-Institut für Innovative Mikroelektronik, Im Technologiepark 25, 15236 Frankfurt, Germany; 2 Semiconductor Materials, BTU Cottbus-Senftenberg, Platz der Deutschen Einheit 1, 03046 Cottbus, Germany

**Keywords:** hexagonal boron nitride, 2D materials, chemical vapor deposition, DFT, borazine

## Abstract

The growth of hexagonal boron nitride (hBN) on epitaxial Ge(001)/Si substrates via high-vacuum chemical vapor deposition from borazine is investigated for the first time in a systematic manner. The influences of the process pressure and growth temperature in the range of 10^−7^–10^−3^ mbar and 900–980 °C, respectively, are evaluated with respect to morphology, growth rate, and crystalline quality of the hBN films. At 900 °C, nanocrystalline hBN films with a lateral crystallite size of ~2–3 nm are obtained and confirmed by high-resolution transmission electron microscopy images. X-ray photoelectron spectroscopy confirms an atomic N:B ratio of 1 ± 0.1. A three-dimensional growth mode is observed by atomic force microscopy. Increasing the process pressure in the reactor mainly affects the growth rate, with only slight effects on crystalline quality and none on the principle growth mode. Growth of hBN at 980 °C increases the average crystallite size and leads to the formation of 3–10 well-oriented, vertically stacked layers of hBN on the Ge surface. Exploratory ab initio density functional theory simulations indicate that hBN edges are saturated by hydrogen, and it is proposed that partial de-saturation by H radicals produced on hot parts of the set-up is responsible for the growth.

## 1. Introduction

Hexagonal boron nitride (hBN) is a two-dimensional (2D) insulator with a wide bandgap of ~5.96 eV and a molecular structure analogous to graphene [1]. It is a promising material for a range of applications, including as a protection layer for high-mobility graphene [2,3,4,5], deep ultraviolet optoelectronics [6,7], solid-state thermal neutron detectors [8], and tunnel barriers in tunnel devices [9,10]. The realization of these devices requires the growth of high-quality hexagonal boron nitride over large areas. Synthesis of hBN films has been realized by a multitude of methods, such as mechanical exfoliation [11,12], molecular beam epitaxy [13,14], ion beam sputtering deposition [15], and co-segregation [16]. However, the most common method so far is chemical vapor deposition (CVD) growth of hBN on catalytic transition metal substrates such as Cu and Ni. Growth of high-quality hBN single and multilayers up to 2” in diameter was recently demonstrated using this technique [17,18,19,20]. Nevertheless, device fabrication requires the transfer of the hBN films onto CMOS-compatible substrates, which severely limits process scalability. Additionally, 2D films transferred from metal substrates are contaminated with metal atoms at concentrations unacceptable for front-end-of-line Si technology integration [21,22,23]. Therefore, direct growth of hBN thin films on CMOS-compatible substrates, such as Si-based substrates, germanium, or dielectrics, is the ideal scenario.

Although this area of hBN growth studies has received less attention compared to the growth on metal substrates, some progress has been reported recently. The growth of nanocrystalline hBN films has been achieved on Si_3_N_4_/Si and SiO_2_/Si [24], as well as on Si(001) substrates [25,26]. Either ammonia borane or boron trichloride and ammonia were used as the precursors, resulting in film thicknesses ranging from a few nanometers to several micrometers. Optimal growth conditions were found to be at growth temperatures of 1100–1200 °C, yielding crystallite sizes of the order of a couple of nanometers, which is still far from the quality achieved on metal substrates. Germanium is a promising substrate material due to its catalytic activity [27] and proven suitability for CVD growth of 2D materials [28,29]. Presently, less than a handful of publications studying the growth of BN on Ge(001) and Ge(110) substrates have been published. All of them report the growth of polycrystalline hBN monolayers via low-pressure CVD using ammonia borane as the precursor [30,31,32]. It was found that hBN can form well-aligned triangular islands with 90° rotational symmetry on Ge(001) and 180° symmetry on Ge(110) [30]. However, neither systematic studies of the influence of the process parameters nor growth mechanisms of BN on Ge have been reported. 

Therefore, the aim of this work was to systematically explore high-vacuum CVD growth of hBN thin films on Ge(001) using borazine as a function of pressure and growth temperature, and to look at possible growth mechanisms via density functional theory (DFT) simulations. Firstly, the impact of the reactor pressure is evaluated with respect to morphology, growth rate, and crystallinity of the hBN films. Secondly, the effect of the growth temperature is studied, mainly focusing on the crystalline quality. Finally, first results of exploratory ab initio density functional theory simulations are presented, pointing to possible growth mechanisms and supporting the interpretation of experimental data.

## 2. Materials and Methods

Growth of hBN films was carried out on epitaxial Ge(001)/Si(001) substrates [33] using a RIBER Compact 21T ultra-high-vacuum (UHV) CVD system. In order to remove the native germanium oxide from the surface, the samples were annealed at 900 °C for 30 min in UHV prior to the growth. Borazine (B_3_N_3_H_6_, Katchem, Prague, Czech Republic) was used as a single-source precursor and supplied to the chamber via a bubbler, using Ar (99.9999 %, Air Liquide, Düsseldorf, Germany) as the carrier gas. The temperature of the bubbler was kept at −10 °C to ensure constant borazine vapor pressure. Borazine was introduced to the chamber at different pressures ranging from 10^−7^ mbar to 10^−3^ mbar for 120 min, at temperatures ranging from 900 °C to 980 °C. After growth, the samples were rapidly cooled to room temperature. It should be noted that the reported temperatures are setpoint temperatures and that the sample surface temperature is at least 50 °C lower, as the Ge surface did not melt at T_SP_ = 980 °C (the melting point of Ge is 938.3 °C).

The chemical composition of the deposited hBN films was investigated by in situ X-ray photoelectron spectroscopy (XPS, SPECS GmbH, Berlin, Germany) using an Al Kα X-ray source. The morphology of the samples was characterized using atomic force microscopy (AFM, tapping mode, Dimension Icon, Bruker, Billerica, MA, USA). Transmission electron microscopy (TEM, 200 kV acceleration voltage, Tecnai Osiris, FEI, Hillsboro, OR, USA) and Raman spectroscopy (532 nm excitation, Renishaw inVia, Renishaw, Wotton-under-Edge, UK) were performed to investigate the crystalline quality of the hBN films. Raman peak positions were calibrated to the Si peak at 520.5 cm^−1^ and the bulk hBN peak at 1366 cm^−1^.

Atomic structures were optimized on the JUWELS cluster [34] by the Quantum Espresso (QE) [35,36] package within the ab initio plane wave pseudopotential density functional theory (DFT). Exchange–correlation energy was computed on the generalized gradient approximation (GGA) [37] level using the PBE form [38]. Van der Waals interactions were included in the non-local meta-GGA form (rVV10) [39,40]. Energy barriers on the reaction paths between atomic structures of interest (as for surface diffusion or for precursor decomposition) were estimated with the Nudged Elastic Band (NEB) technique [41] and refined with the Climbing Image (CI-NEB) method [42].

The calculations for isolated molecules were performed in cubic supercells (34 Bohr or 40 Bohr), and the Makov–Payne correction [43] was used to impose their isolation across the surrounding vacuum. Hexagonal BN was described by either a monolayer (supercell size 6 × 6, or 36 atoms) or a bilayer (72 atoms) film, whereby the bottom layer was kept fixed during relaxation of the remaining atoms. The supercell size in the direction normal to the hBN surface was 40 Bohr for monolayer and 60 Bohr for bilayer slabs, and the spurious electrostatic interaction across the vacuum was removed by the effective screen medium (ESM) method [44]. The Brillouin zone was sampled at the Γ point of the 12 × 12 surface supercell, which yielded converged results. The Ge(001) surface was simulated by a 4 × 4 surface supercell with 2 × 2 buckled dimers [45]. The slabs contained eight (001) atomic layers, whereby the bottom two were fixed at bulk positions and the bottom surface was saturated with hydrogen.

## 3. Results and Discussion

**Figure 1:**
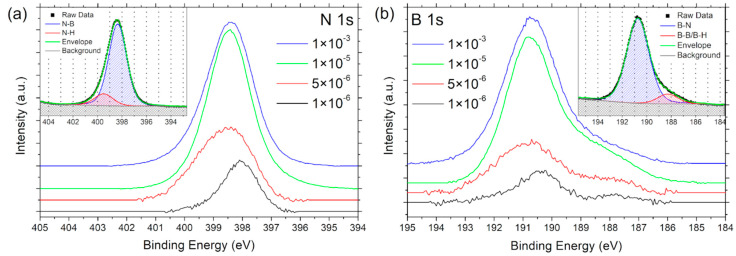
(**a**) N 1s and (**b**) B 1s XPS spectra of hBN films grown on Ge(001) at various pressures (in mbar). Insets show peak fit model with two components and Tougaard background, exemplified for 1 × 10^−3^ mbar.

To investigate the influence of the reactor pressure on the hBN growth, samples were prepared at 1 × 10^−6^ mbar, 5 × 10^−6^ mbar, 1 × 10^−5^ mbar, and 1 × 10^−3^ mbar, at a growth temperature of 900 °C. Growth experiments at 1 × 10^−7^ mbar yielded only trace amounts of non-stoichiometric boron nitride and were not considered further. The chemical composition of the deposited boron nitride films was investigated by in situ XPS. The N 1s and B 1s spectra are displayed in Figure 1, indicating the growth of boron nitride for all pressures. At 1 × 10^−6^ mbar, the N 1s spectrum exhibits two overlapping peaks at 398.3 eV and 399.4 eV, which can be attributed to N–B and N–H bonds, respectively, in agreement with literature values [46,47]. For peak fitting, Gaussian–Lorentzian line shapes and a Tougaard background were used for all spectra. The B 1s spectrum can be fitted with two peaks: one at 190.8 eV corresponding to B–N bonds [46], and one at 187.9 eV corresponding to B–B [48] or possibly B–H bonds, as boron and hydrogen have similar electronegativity values. This peak model (also shown in the insets of Figure 1) was used to fit the XPS spectra of all samples. The presence of N–H and B–H bonds can be explained by incomplete dehydrogenation of borazine during growth. B–B bonds could indicate the formation of large numbers of metallic grain boundaries, B–B point defects, or B clusters, all of which are plausible considering the results of first ab initio DFT calculations described at the end of this section. N–N bonds (406.0 eV) [49], on the other hand, were not observed. The N:B ratio is 1 ± 0.1 for all samples.

**Figure 2:**
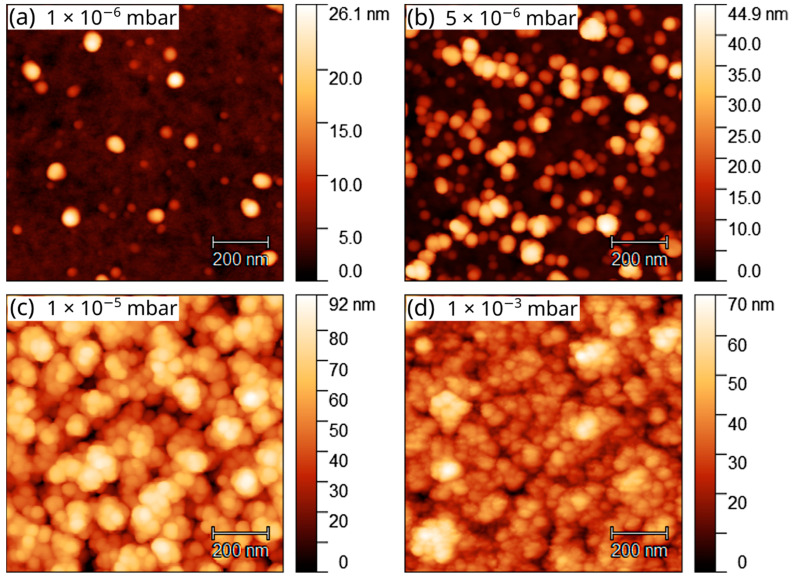
AFM images of hBN samples grown on Ge(001) at (**a**) 1 × 10^−6^ mbar, (**b**) 5 × 10^−6^ mbar, (**c**) 1 × 10^−5^ mbar, and (**d**) 1 × 10^−3^ mbar.

With increasing pressure, the intensity of the XPS signal increases at first and then saturates at 1 × 10^−5^ mbar. This effect is attributed to an increasing coverage of the Ge surface with three-dimensional boron islands, as shown by AFM (see Figure 2). At 1 × 10^−5^ mbar, the substrate surface is completely covered, leading to no further increase in intensity as the film thickness exceeds the information depth of XPS (3λ ≈ 3 nm in hBN) [50]. Additionally, with increasing pressure, a shift towards higher binding energies is observed for both the N 1s and B 1s spectra, which is consistent with positive charge buildup on the surface of the insulating boron nitride film during the XPS measurement, due to the increasing coverage of the conductive substrate. This finding is further supported by the fact that all peaks shift by the same amount in both the N 1s and B 1s spectra, so that their separation remains constant, which indicates unchanged chemical states. Due to the in situ nature of the measurement suppressing carbon contamination, the widespread practice of charge referencing to the C 1s spectrum of adventitious carbon (to mitigate the shift) was not applied.

The morphology of the samples was investigated with AFM. Figure 2 shows the AFM images with a scan size of 1 × 1 µm^2^, acquired in tapping mode. At low pressure (Figure 2a), three-dimensional islands are formed, measuring up to 80 nm in diameter and 22 nm in height. As the pressure increases (Figure 2b–d), nucleation density and growth rate increase, leading to the formation of a continuous film at ~1 × 10^−5^ mbar. RMS roughness of the sample surface increases from 2.7 nm at 1 × 10^−6^ mbar to 9.1 nm at 5 × 10^−6^ mbar and 15.3 nm at 1 × 10^−5^ mbar, then decreases again to 10.2 nm at 1 × 10^−3^ mbar.

**Figure 3:**
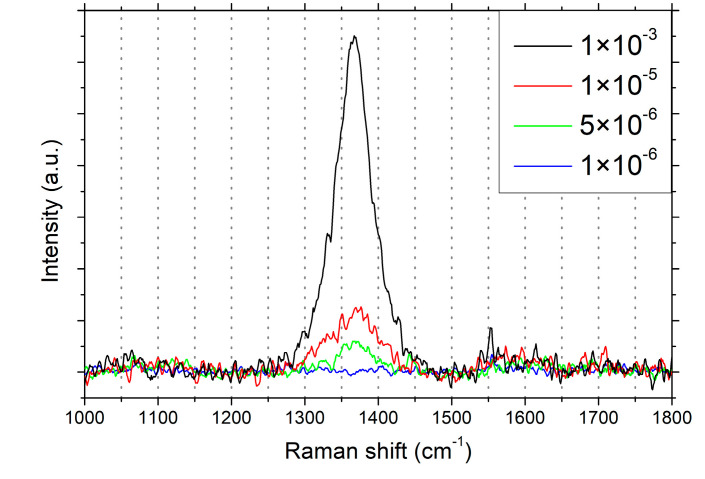
Raman spectra of hBN films grown on Ge(001) at various pressures (in mbar).

Raman spectroscopy was performed to determine the crystallinity of the deposited boron nitride films; the spectra are displayed in Figure 3. Starting at 5 × 10^−6^ mbar, the characteristic E_2g_ phonon mode of hBN is observed, confirming the presence of a hexagonal BN phase in the deposited films. The intensity of the peak increases with increasing pressure, indicative of either increasing crystallinity or increasing film thickness, as Raman intensity is proportional to the number of layers in hBN [51]. The peak position is shifted towards a higher frequency compared to bulk hBN (1366 cm^−1^) [52] at 5 × 10^−6^ mbar and 1 × 10^−5^ mbar: to 1369.8 ± 1.5 cm^−1^ and 1367.5 ± 0.8 cm^−1^, respectively. At 1 × 10^−3^ mbar, the peak is centered at 1366.5 ± 0.2 cm^−1^, close to the bulk value. The position of the Raman peak of hBN is influenced by several mechanisms: small crystallites were found to induce a shift towards higher frequencies [53], and even small amounts of strain can induce a large shift in either direction, depending on its sign [54]. Strain, in turn, can be a result of substrate roughness or difference in thermal expansion coefficient. Considering our results, we expect a mixture of these effects, especially for the film grown at 1 × 10^−3^ mbar, as the peak position is close to the bulk value, whereas the width of the peak is much larger than expected for bulk hBN. 

Generally, the Raman peaks of our hBN films are broad, with a full width at half maximum (FWHM) of 47.0 ± 3.5 cm^−1^, 77.0 ± 1.9 cm^−1^, and 59.1 ± 0.6 cm^−1^ for 5 × 10^−6^ mbar, 1 × 10^−5^ mbar, and 1 × 10^−3^ mbar, respectively, suggesting a nanocrystalline structure of the hBN films. Similar values were recently reported for hBN films grown on other non-metal substrates, including Si(001) and sapphire [25,26], while there are no reports of FWHM values for Ge substrates to date. Evidently, the crystalline quality of hBN on metal-free substrates is still subpar compared to films grown on transition metal substrates; for example, a FWHM of 14.5 cm^−1^ was reported for hBN monolayers grown on Cu(111) [20].

**Figure 4:**
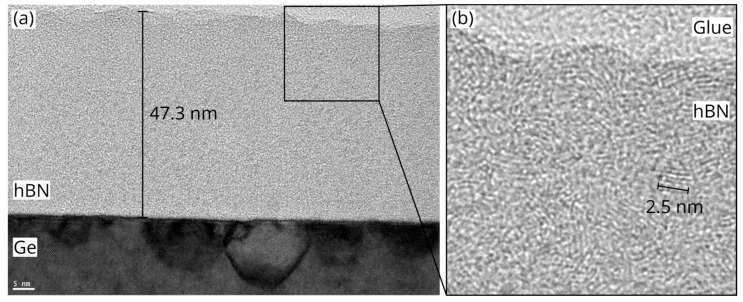
(**a**) Cross-section TEM image of hBN film grown on Ge(001) at 900 °C and 1 × 10^−3^ mbar. The scale bar is 5 nm. (**b**) Zoomed in view of the area indicated in (**a**) by the black square.

Raman FWHMs can be used to calculate the approximate lateral sizes of hBN crystallites via the Nemanich model [53]:

$$L_{a}=1417/\left(\Gamma_{1/2}-8.7\right),\;$$

where $L_{a}$ is the crystallite size in Å and $\Gamma_{1/2}$ is the FWHM in cm^−1^. Using the FWHM values stated above, the lateral crystallite sizes of the hBN films are 3.7 nm, 2.1 nm, and 2.8 nm. Similar values were recently reported for hBN films grown on sapphire [25], while large-scale, single-crystalline films have been achieved on Cu substrates [20,55] and molten gold [56]. The crystallinity of the hBN film grown at 1 × 10^−3^ mbar was further investigated with cross-section TEM. As shown in Figure 4, the film is about 47 nm thick and exhibits a nanocrystalline structure with many randomly oriented crystallites 2–3 nm in size. These findings are in line with the results obtained by Raman spectroscopy. It should be noted here that the abovementioned equation is an empirical model, fitted for an unknown distribution of crystallite sizes in the original samples, as acknowledged by the authors [53]. Thus, it should only be seen as an approximation for all other samples. Additionally, analogous to the argumentation of Oliveira et al. for the case of graphene [57], the model most likely underestimates the crystallite size of any given sample. Both point defects and the shift of the Raman peak (which is dependent on crystallite size and strain) additionally contribute to the broadening of the peak. Consequently, the calculated values of $L_{a}$ can be considered as the lower limits of the lateral crystallite size.

**Figure 5:**
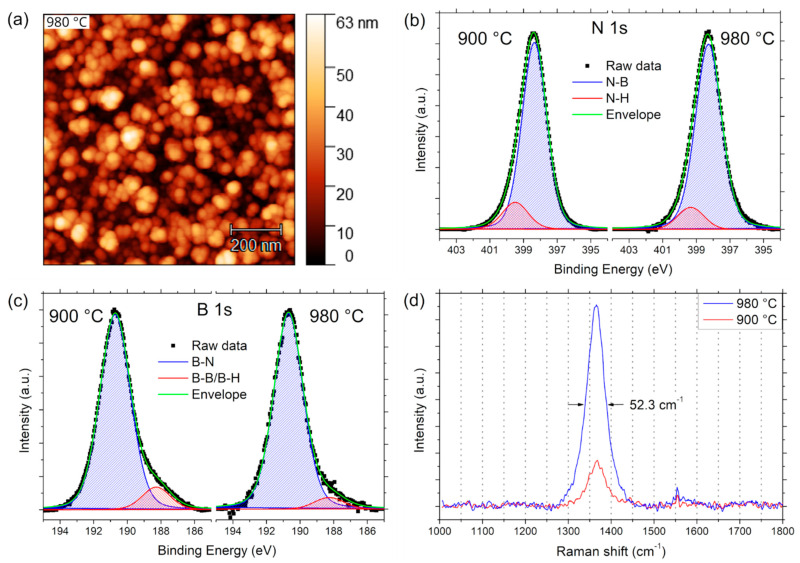
hBN films grown at different temperatures. (**a**) AFM image of sample grown at 980 °C; (**b**) XPS N 1s spectra (Tougaard background subtracted); (**c**) XPS B 1s spectra; (**d**) Raman spectra.

**Figure 6:**
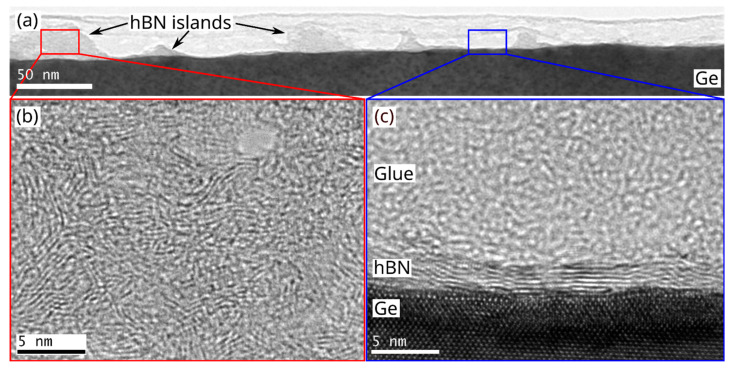
(**a**) Overview TEM image of sample grown at 980 °C, showing islands and flat regions; (**b**) detailed view of island marked by red box in (**a**); (**c**) detailed view of vertically stacked hBN layers between islands marked by blue box in (**a**).

After performing the growth experiments at various pressures, the growth temperature was increased to 980 °C in order to influence the crystalline quality of the hBN films, while the pressure was set to 1 × 10^−3^ mbar. The samples were investigated by AFM, XPS, Raman, and TEM, as shown in Figure 5 and Figure 6. It was found that the surface morphology (Figure 5a) and RMS roughness (12.0 nm), as measured by AFM, did not change significantly. However, XPS analysis of the N 1s and B 1s regions (Figure 5b,c) shows that the contribution of the B–B/B–H bonds is reduced compared to the sample grown at 900 °C, after fitting with the aforementioned two-component model with Tougaard background. 

This is a first indication of improved crystalline quality after increasing the growth temperature to 980 °C, which was then confirmed by Raman spectroscopy, as shown in Figure 5d. The intensity of the peak centered at 1366.5 ± 0.1 cm^−1^ is increased and the width is reduced, with a FWHM of 52.3 ± 0.3 cm^−1^ (down from 59.1 ± 0.6 cm^−1^ for the sample grown at 900 °C). The approximate lateral crystallite size according to the Nemanich model is increased to 3.3 nm.

The improved quality becomes more apparent in the cross-section TEM images shown in Figure 6. In the overview image (Figure 6a), two different regions can be identified: (i) island regions (Figure 6b), consisting of randomly oriented nanocrystalline hBN features similar to the ones grown at 900 °C, but with an increased crystallite size of 2–5 nm, and (ii) vertically stacked hBN regions between the islands (Figure 6c), where ordered growth of hBN layers can be observed. The number of these vertically stacked layers varies between 4 and 10 layers across the sample, with an interlayer distance of 3.35 Å, close to the literature value of 3.33 Å [58]. Unfortunately, it was not possible to further increase growth temperature or pressure, due to technical limitations. Additionally, the comparatively low melting point of Ge provides a natural upper limit on practicable growth temperatures. Nevertheless, the results obtained at 980 °C are promising and might be further improved by the addition of H_2_ to the process, which will be investigated in the future.

**Figure 7:**
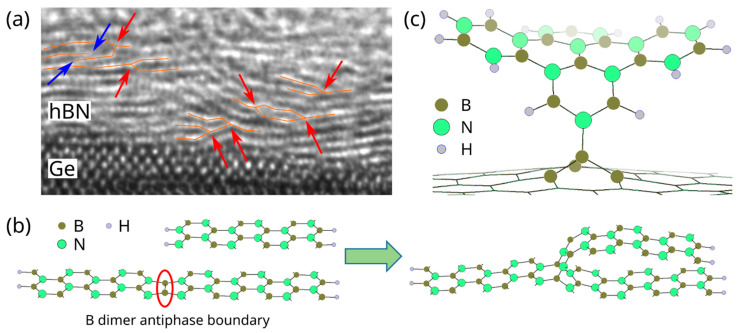
(**a**) Cross-section HRTEM image showing multiple bifurcations (red arrows), possibly as a product of the process shown in (**b**), as well as two detached layers (blue arrows). The hBN layers have been partially highlighted as a guide to the eye. (**b**) Initial state of separate, parallel hBN ribbons and relaxed final state of hBN ribbons attached at the former B dimer antiphase boundary. (**c**) Nucleation of a new hBN layer at a B_N_ point defect.

Finally, first ab initio DFT/NEB simulations were performed in order to elucidate possible explanations for certain structures observed in the vertically stacked hBN regions via HRTEM. Only a short overview of first results is given here; a more complete and detailed study will be published elsewhere. The hBN layers appear to be frequently interconnected, forming bifurcation-like structures, as shown in Figure 7a (indicated by red arrows). It was found that two parallel hBN ribbons can connect at B dimer antiphase boundaries and that the connected ribbons then relax to form bifurcation-like structures. The process is shown schematically in Figure 7b. In addition to providing an attachment point for an already formed hBN layer, B dimer antiphase boundaries could possibly serve as a nucleation point for new hBN layers during growth, resulting in the same structure. This process can thus be seen as a possible origin of the observed bifurcations. Similarly, substitutional boron point defects were found to be nucleation sites for new hBN layers that start out perpendicular to the original layer, as shown in Figure 7c. These new layers are expected to detach as they grow, because they are anchored with only a single chemical bond. In our HRTEM images, we observe some hBN layers that seem to just end without being attached to any of the neighboring layers, as indicated by blue arrows in Figure 7b, which might be a product of this nucleation and detachment process. Both B dimers and B_N_ point defects are sources of B-B bonds, which were observed via XPS, lending further credibility to these findings.

Another possible source for B-B bonds, however, could be B clusters formed from atomic boron. Additional DFT simulations were carried out to determine whether decomposition of the borazine molecule down to single atoms is possible on germanium and hBN surfaces, as this would be the only way to obtain atomic B in our experimental setup. They show that full decomposition is indeed feasible on Ge(001). The reaction is endothermic with an energy loss of 5.5 eV, which is compensated by a large entropy gain. A rough estimate from lattice gas entropy indicates that equilibrium with 10^−3^ mbar of borazine precursor is reached at 900 °C when about 0.1% of the surface sites are covered by B, N, and H atoms. The energy barriers to break a BN bond are higher than the barriers for dehydrogenation, but may be as low as 1.9 eV (scission of an H-free BN dimer), so that at 900 °C, a BN bond may open within microseconds. This means a significant number of these reactions is expected to take place at our experimental conditions and provide atomic B, N, and H species. The formation of B clusters from atomic B is therefore plausible on the Ge(001) surface, but due to the low information depth of XPS, their contribution to the B 1s spectrum should be negligible, except in the case of very low pressure or growth temperature. On the other hand, we expect no B clusters in the top layers of thicker films, because the aforementioned borazine decomposition should be inefficient on the hBN surface. Firstly, we found that adsorption of borazine on perfect, intrinsic hBN is low, with a desorption energy of 0.5 eV. This means that, assuming that the diffusion of borazine molecules is ballistic at thermal velocity, in accordance with ab initio estimates, and that the vibrational frequency perpendicular to the surface is 2 × 10^13^ Hz, the life time in the adsorbed state is of the order of 10 ps at 900 °C. In that time, the average molecule visits only about ten surface sites before it desorbs, so that adsorption and diffusion do not strongly enhance the flux of molecules available to surface reactions [59]. Secondly, the decomposition reactions are energetically unfavorable, with an energy loss of 4.3 eV just for detachment of one H atom. As they also come with very little change in the gas lattice entropy, decomposition of borazine on hBN should be negligible.

As a consequence, these findings suggest that hBN growth should proceed only at the edges of the hBN film and from intact borazine molecules, once the Ge surface has been covered by hBN. Hence, further NEB simulations explored possible mechanisms and challenges of this edge growth. They show that direct bonding of borazine molecules to H-terminated hBN edges is inefficient, leading to very low growth rates. This is in accordance with our experimental results (1–3 nm of well-ordered growth in 2 h). The limiting step is the desorption of the excess H_2_ molecule, which occurs with an energy barrier of 2.0 eV. This means that at 900 °C and 1 mbar of borazine (several orders of magnitude higher than our experiments), a lateral growth rate of only 25 nm/h (from each edge) is expected from this mechanism. This is too slow to explain our results, but the reaction is much faster if the hBN edge site is free of hydrogen. In that case, the energy barrier is only 0.05 eV and the reaction is accompanied by an energy gain of about 0.7 eV, providing an easy pathway for hBN growth. The excess H atom from the borazine molecule can then easily desorb with a barrier of only 0.09 eV. We found that an efficient way to remove the H atom from the hBN edge is via atomic H, which can be produced from H_2_ gas at hot surfaces inside the reaction chamber, such as the sample holder or the heater itself. Some amount of H_2_ gas is always present in the reaction chamber, as a product of other reactions involving the borazine molecule. Nevertheless, it is expected that supplying additional H_2_ gas would indeed positively impact the growth process. In addition, there might be further processes contributing to the growth of hBN from borazine which require further investigation.

## 4. Conclusions

The growth of hexagonal boron nitride films via chemical vapor deposition from borazine on epitaxial Ge(001)/Si substrates was investigated within the pressure range from 10^−7^ mbar to 10^−3^ mbar and at setpoint temperatures of 900 °C and 980 °C. At 900 °C, boron nitride was successfully deposited starting at 1 × 10^−6^ mbar, and the presence of the hexagonal phase was confirmed by Raman spectroscopy starting at 5 × 10^−6^ mbar. The films exhibit a nanocrystalline structure with average crystallite sizes ranging from 2.1 nm to 3.7 nm, as calculated from the FWHM of the Raman peaks and confirmed via cross-section TEM. Increasing the pressure beyond 5 × 10^−6^ mbar mainly led to an increasing growth rate, with only a small impact on the crystalline quality. AFM revealed a morphology dominated by islands up to 80 nm in width, demonstrating three-dimensional growth of the hBN films. The crystalline quality of the films can be improved by increasing the growth temperature. At 980 °C, the average crystallite size increased and the formation of 4–10 well-oriented, vertically stacked hBN layers at the Ge/hBN interface, extending between the nanocrystalline islands, was observed by cross-section TEM. Finally, exploratory ab initio DFT/NEB simulations showed that additional hBN layers can nucleate at B dimer antiphase boundaries, leading to bifurcation-like structures, as observed via TEM. Furthermore, it was found that the Ge(001) surface facilitates borazine decomposition to single atoms, while perfect hBN does not, and that efficient growth requires the presence of H radicals. In conclusion, our work provides a solid groundwork for a better understanding of the growth processes of hBN on germanium, which we believe is one of the most promising CMOS-compatible substrate materials for hBN. From this foundation, further questions, such as the benefit of adding H_2_ gas to the process and the exact nature of borazine fragments contributing to the growth, as well as further improvement of the crystalline quality, can be addressed in the future.

## Data Availability

The data that support the findings of this study are available from the corresponding author upon reasonable request.
